# Impact of Stress and Financials on Romanian Infertile Women Accessing Assisted Reproductive Treatment

**DOI:** 10.3390/ijerph19063256

**Published:** 2022-03-10

**Authors:** Roxana Margan, Madalin-Marius Margan, Corneluta Fira-Mladinescu, Salomeia Putnoky, Ioana Tuta-Sas, Radu Bagiu, Zoran Laurentiu Popa, Elena Bernad, Ioana Mihaela Ciuca, Felix Bratosin, Oana Codruta Miloicov-Bacean, Brigitha Vlaicu, Amadeus Dobrescu

**Affiliations:** 1 Department 14 Microbiology, Discipline of Hygiene, Center for Studies in Preventive Medicine, “Victor Babes” University of Medicine and Pharmacy, 300041 Timisoara, Romania; marganroxana@gmail.com (R.M.); fira-mladinescu.corneluta@umft.ro (C.F.-M.); putnoky.salomeia@umft.ro (S.P.); tuta-sas.ioana@umft.ro (I.T.-S.); bagiu@umft.ro (R.B.); baceano@gmail.com (O.C.M.-B.); vlaicu@umft.ro (B.V.); 2 Department of Microscopic Morphology, “Victor Babes” University of Medicine and Pharmacy, 300041 Timisoara, Romania; 3 Department of Obstetrics and Gynecology, “Victor Babes” University of Medicine and Pharmacy, 300041 Timisoara, Romania; zoranpopal@yahoo.com (Z.L.P.); ebernad@yahoo.com (E.B.); 4 Department of Pediatrics, “Victor Babes” University of Medicine and Pharmacy, 300041 Timisoara, Romania; ciuca.ioana@umft.ro; 5 Department of Infectious Diseases, “Victor Babes” University of Medicine and Pharmacy, 300041 Timisoara, Romania; felix.bratosin7@gmail.com; 6 Department of General Surgery, “Victor Babes” University of Medicine and Pharmacy, 300041 Timisoara, Romania; dobrescu.amadeus@umft.ro

**Keywords:** assisted reproductive treatment, female infertility, access to treatment, psychological impact

## Abstract

Around 20% of couples worldwide are affected by infertility issues, with numbers in the European Union reaching as high as 25%, while access to reproductive care varies significantly by geopolitical and country-specific variables. The purpose of this research is to shed light on the unique social, psychological, and financial difficulties faced by Romanian couples seeking access to assisted reproductive therapy (ART). A cross-sectional study was conducted between 2017 and 2019 to involve women who accessed ART at fertility clinics in Romania by completing two infertility surveys. We analyzed the data in terms of all facets of infertility and ART, including the effect of personal background and stress levels on succeeding to conceive, the impact of treatment costs, and household income. A total of 829 participants completed the survey. We observed that high stress exposure leads to a substantially higher duration to conceive using ART, although the proportions of successful pregnancies did not differ between low-stress and high-stress groups. A significantly higher number of couples achieved pregnancy when their monthly household income was higher than EUR 1000 and if the ART method was more expensive. Additionally, we observed that advanced age, high stress levels, and the high cost of ART had a negative association with achieving pregnancy using ART. The findings indicated that Romanian couples experiencing infertility must contend with significant expenses for specialist infertility treatments, as well as treatment-related stress, both of which have a detrimental effect on their odds of attaining conception.

## 1. Introduction

Infertility affects around one in every six couples of reproductive age globally and more than 25 million inhabitants of the European Union (EU) [1]. The World Health Organization (WHO) acknowledges that despite the high frequency of infertility, the majority of infertile women remain silent about their experience, increasing their psychological fragility. Natural infertility may result in emotions of shame, remorse, and poor self-esteem. These negative emotions might manifest as despair, worry, discomfort, and a low quality of life in varied degrees [2,3]. Other studies report infertility as causing depression comparable to cancer and other life-threatening diseases [4]. On top of the negative psychological effects, infertility still causes stigmatization in couples that are unable to conceive, including the pressuring families and social pressure of peers, disturbing the quality of life, social position, and causing serious relationship tension [5,6]. Existing research indicates that infertility has a greater impact on women than on males, with some women becoming victims of spousal abuse, economic distress, and social isolation [7]. It was observed in Europe that couples seeking assisted reproductive therapy (ART) are more likely to have a higher socioeconomic status than the general population [8], since couples from this category often elect to have children later in life, after achieving career goals [9].

Options regarding infertility treatment vary nowadays by complexity, success rates, and, consequently, their costs. Assisted reproductive therapy (ART) in Romania has evolved extensively during the past two decades, offering a range of procedures, including in-vitro fertilization (IFV) and embryo transfer, intracytoplasmic sperm injection (ICSI), gamete intrafallopian transfer, zygote intrafallopian transfer, tubal embryo transfer, gamete and embryo cryopreservation, oocyte and embryo donation, and gestational surrogacy [10,11]. It is estimated that at least 8 million babies have been born through these assisted techniques since the first successful attempt [12], although the numbers in Romania remain unclear since most of them are performed in private practices.

Even though modern and expensive reproductive methods offer a statistically higher rate of success, several modifiable and unmodifiable factors are known to influence or might influence these numbers. While some lifestyle factors, such as cigarette smoking, illicit drug use, and alcohol and caffeine consumption, can be detrimental to female fertility, others, such as preventative care, can be favorable. Among the strongest impact factors on fertility as described by the scientific literature are the patient BMI, stress exposure, abnormal reproductive organ anatomy, and delayed childbearing age of starting a family, the latter two being unmodifiable [13,14]. Women experiencing infertility overwhelmingly believe that stress plays a role in their failure to conceive [15], and those who seek ART are particularly worried that stress may lower their chances of getting pregnant [16,17]. Multiple mechanisms by which the stress exposure and depression may influence female fertility were suggested; however, populational studies on fertility with this main goal are scarce [18]. This has resulted in imprecise conclusions about the effect of depression on infertility [4,19], although several published studies have indicated that infertile women receiving fertility treatment experience higher levels of stress and a higher prevalence of depression and anxiety than the general population [20,21]. Additionally, these symptoms are more prevalent in individuals who have had many IVF rounds after unsuccessful efforts [22,23].

In light of the aforementioned information, we sought to address the stress and financial factors that are most prevalent in Romania, and represent a susceptible reason for impeding access of infertile couples to assisted reproductive treatment in our country.

## 2. Materials and Methods

The present study was conducted between 2017 and 2019, and took place in an outpatient setting, in a joint collaboration between five fertility clinics from university medical centers in Romania, affiliated with the “Victor Babes” University of Medicine and Pharmacy Timisoara. The study was built on a cross-sectional design, and we opted for a convenience sampling technique to calculate the optimal sample size, which was estimated based on the prevalence of infertile couples to include at least 245 individuals for a confidence coefficient of 95%, and a 5% margin of error.

The eligibility criteria comprised women of reproductive age from couples with a history of at least 12 months of being unsuccessful attempting pregnancy. All respondents completed the ART treatment, and were later requested by their doctors to answer a survey developed by the researchers to assess their overall experience. The questionnaire was also made accessible online for simplicity of use and to ensure completion and submission of the questionnaire. Participants provided informed permission for data processing after being guaranteed of confidentiality of personal identifiers. The questionnaire was divided into eight parts and included 65 questions on social and financial factors, such as current yearly family income, degree of education, and amount invested by couples in ART. Another factor considered important to survey was the intercourse pattern, where participants were assessed on a three-point Likert scale: rarely = “once a month”, often = “once a week”, frequently = “more than once a week”. Patients were interrogated for partnership status (married or unmarried) and support (feeling like being/not being supported by the partner for infertility issues). The stress level experienced by women undergoing ART was measured since the beginning of treatment, and quantified on a four-point Likert scale: 1 = “rarely experiencing stress”, 2 = “sometimes experiencing stress”, 3 = “often experiencing stress”, 4 = “very often experiencing stress”. Based on stress levels determined by surveying our participants, we subdivided them into two groups, “low stress exposure” and “high stress exposure”. Those who experienced stress rarely or sometimes were considered the control group, while those who were confronted with stress often and very often were considered as cases. A total of 829 eligible women with a history of infertility successfully completed the surveys.

The Local Committee of Ethics for Scientific Research of the “Victor Babes” University of Medicine and Pharmacy Timisoara operates under art. provisions 167 of Law no. 95/2006, art. 28, chapter VIII of order 904/2006, and with EU GCP Directives 2005/28/EC, International Conference on Harmonization of Technical Requirements for Registration of Pharmaceuticals for Human Use (ICH), and with the Declaration of Helsinki—Recommendations Guiding Medical Doctors in Biomedical Research Involving Human Subjects. The current study protocol received ethical approval on 7 April 2017, with approval number 4235. All patients included in the study agreed to be involved by signing a standardized informed consent form.

Our data were analyzed using the SPSS v.26 software (IBM, Chicago, IL, USA) for Windows operating system, performing the Chi-Square and Fisher’s test to analyze proportions, the Kruskal–Wallis and Mann–Whitney U-test to compare mean ranks, and Spearman correlation was calculated to observe significant associations between the studied variables. A multivariate analysis was performed to determine the factors associated with the risk of failing to achieve pregnancy. The significance threshold was α < 0.05.

## 3. Results

The mean age of women was 32, ranging from 24 to 44 years old, where 86.6 percent of them were married and the rest were in a relationship. Our survey discovered that more than 84 percent of the study participants were working, 6.5 percent were self-employed, and 8.7 percent were jobless. We observed that more than 38.6 percent of couples sought natural conception for fewer than five years, but the majority, at 48.2 percent, struggled to conceive for between five and ten years. The remaining 13.2 percent of couples endured a prolonged duration of infertility, lasting more than ten years. Concerning pregnancy outcomes, 56.5 percent of women did not become pregnant, and only 10.6 percent became pregnant spontaneously; 3.2 percent became pregnant following ovarian stimulation, and a significant 29.6 percent became pregnant following intrauterine insemination (IUI)/in-vitro fertilization (IVF)/gamete intrafallopian transfer (GIFT). Concerning the effectiveness of assisted reproductive treatments, 56.1 percent of women who achieved a pregnancy via ART did so through IVF, 22% via IUI, and just 3.3 percent via GIFT.

A total of 307 (37.0 percent) of the 829 female respondents did not report being exposed to high levels of stress, whereas 420 (63.0 percent) reported experiencing high levels of stress (Table 1). Consequently, continuous exposure to stress might be deemed a significant risk factor for the prolonging of infertility (*p* = 0.012), and the 95% confidence interval for OR = (1.29; 3.47). The time period between unsuccessful conception and pregnancy was considerably longer for individuals who were often exposed to stress (8.83 years vs. 7.87 years) (Mann–Whitney test, *p*-value = 0.021). Additionally, we examined the relationship among both stress and academic achievement by classifying the results into a higher education group consisting of those who achieved a college, masters, or PhD level qualification. The lower education group consisted of women who achieved a maximum of a high school diploma. The findings indicate that the proportion of women exposed to stress is considerably higher among those with less education (Chi-Square test *p*-value = 0.011).

Compared with 463 women who were successful in conceiving, a total of 343 women failed to achieve pregnancy, but the majority were not exposed to high levels of stress, and the difference was not statistically significant (79.1% vs. 81.4%, Chi-Square test *p*-value < 0.393). We observed that patients are facing significantly lower levels of stress when their partner is supportive, compared with the opposite (Mann–Whitney test, *p*-value < 0.001). Another important factor affecting stress levels in women with infertility was the household income, where 62.2% couples with a household income lower than EUR 1000 complained of feeling higher levels of stress, compared with only 16.6% of couples with an income higher than EUR 1500 (Chi-Square test *p*-value < 0.001) (Table 1). Similar differences in stress levels were determined by the government coverage of ART treatment. A total of 667 (82.5%) of couples who answered our questionnaire responded that they received no government coverage, and 69.4% of them felt under high stress (Chi-Square test *p*-value < 0.001). The relationship between stress levels and expenditure is statistically significant (Chi-Square test *p*-value = 0.010). Another piece of evidence regarding financial aspects of ART is the stress level experienced by patients, since the stress level experienced by those who spent between 4 thousand and 12 thousand euros was significantly higher than in patients who had to spend a maximum of 4000 euros to benefit from ART.

In Table 2, we describe the financials interfering with the success of ART. A statistically significantly higher number of couples with a high household income achieved pregnancy. The disparities in monthly mean earnings per family and the value of treatment investments are significant (Chi-Square test, *p*-value < 0.001), whereas the proportion of patients with an average monthly income greater than 1200 euros and expenses less than 1000 euros is statistically significantly inferior to the distribution of women with a monthly income under 1200 euros (*p*-value < 0.001). Our analysis showed that a significant proportion of couples (30.4 percent) spend more than 4500 euros for ART; 28.6% paid between 1000 and 2500 euros, 28.1% had costs for ART access of less than 1000 euros, and the remaining 12.9% of couples paid between 2500 and 4500 euros. The difference in proportions in the cost of ART was statistically significant when comparing couples who achieved pregnancy and those who did not (*p*-value = 0.022). Lastly, by evaluating the government coverage of ART costs, there were no significant differences in the proportions of couples who succeeded in achieving pregnancy and those who did not (*p*-value = 0.385).

**Figure 1:**
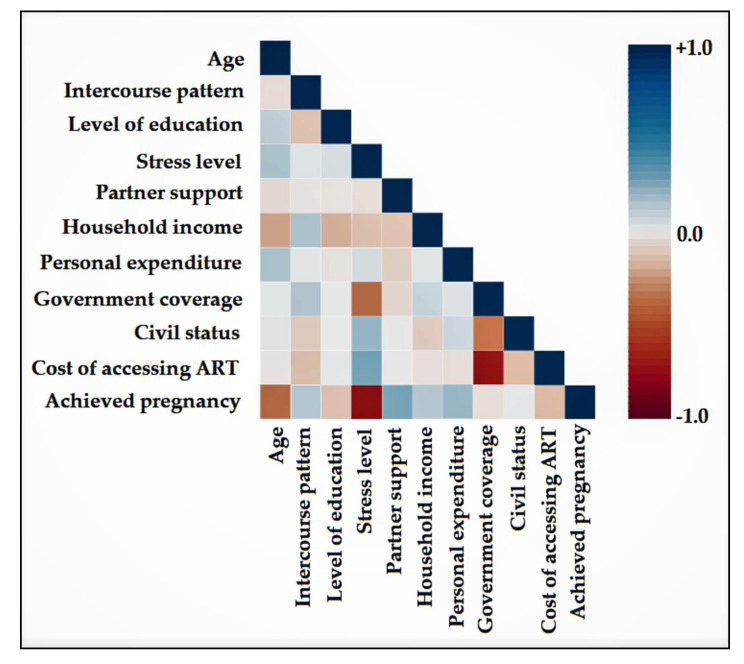
Correlation matrix of variables mentioned in the couple infertility questionnaire. Negative Spearman’s correlation coefficients represented in tones of red color. Positive Spearman’s correlations are represented in tones of blue color.

The correlation analysis shown in Figure 1 had the purpose of determining the variables that are highly associated with succeeding in achieving pregnancy after ART methods. It was determined that age, level of stress, level of education, and cost of accessing ART were negatively associated with a couple with infertility issues being able to conceive. However, only age and stress level were statistically significant (*p*-value = 0.046, respectively, 0.002). The variables with a significant positive correlation with achieving pregnancy were partner support and personal expenditures (*p*-value = 0.010, respectively, 0.007).

The risk factor analysis determined that age was the largest independent risk factor for failing to achieve pregnancy through ART (OR = 2.97, *p*-value = 0.008). Other independent risk factors are presented in Table 3. In ascending order, risk factors start from not having partner support, high costs of accessing ART, low personal expenditures, high level of stress exposure, and lastly, the female’s age.

## 4. Discussion

We discovered that over 80% of the women in our study had a bachelor’s degree or a master’s or doctoral degree, indicating that education is important and may have an effect on how women approach infertility issues. In population-based research, a higher degree of education was related to a reduction since it is critical in recognizing the detrimental consequences of hazardous sexual conduct or an imbalanced lifestyle on fertility [24,25]. A second factor was postponing childbirth, since higher educational objectives may be jeopardized by the time commitment required for both parenting and studying [26]. Additionally, the financial effect of infertility treatments might affect a person’s ability to continue their education [27]. Surprisingly, similar to our findings, investigations done in Scotland revealed no correlation between greater education and infertility [28]. These disparate findings are the consequence of substantial spatial variance in socioeconomic disparities linked with failure to achieve a pregnancy and access to ART. The present study clearly shows that people with a lower level of education are substantially more likely to be subjected to stress than those with a greater level of education. This suggests that women with a university degree are more knowledgeable on the subject of infertility and the possibility of achieving a pregnancy through assisted reproductive treatments and hence do not experience the same amount of stress as women without a university degree.

Two cohorts of a hundred infertile and a hundred fertile couples were used in a Chinese study examining the psychological effects of infertility, with the major end measure being the impact on quality of life. After evaluating the data, it was observed that the infertile group, independent of their marital status, had a poor quality of life [29]. Considering the premises of poor quality of life in this population, several studies suggest that the more worried women are before and throughout infertility treatment, the lower their pregnancy rates become [30,31], although different observations were made by other researchers that concluded the opposite, after discovering no correlation between the mental discomfort associated with reproductive issues and future chances of pregnancy [32,33].

In our research, we discovered that women who do not conceive are more worried, as are those who do not receive support from their spouses throughout this procedure. Disappointment with the prospect of pregnancy has a strong correlation with stress and despair. There is a strong correlation between managing stress and the costs associated with achieving a pregnancy. Subsequently, we discovered that women who spent under 1000 euros were less stressed than those who invested a sum included in the interval 1000–2500 euros. Contributing more money in this process leads to higher expectations that must be satisfied; consequently, as they invest more money, they suffer more stress. In Romania, where the average monthly salary after taxes is around RON 3000, or 625 euros [34], a large number of couples spend a remarkable sum of over 4500 euros on treatment. In the effort to access expert reproductive therapies, couples endure massive financial sacrifices.

Furthermore, the situation in which the vast majority of couples find themselves being responsible for covering the expenses of therapy adds to the stress. Our analysis discovered that just 0.6 percent of all patients had their whole healthcare costs reimbursed by the National Health Insurance Program, and just 16.4 percent had their interventions and therapies partly covered by the Health Insurance Program. Couples’ expectations grow when they spend their own money to obtain a pregnancy, and each failure is accompanied with the potential of investing even more money in the future, with no assurance of success. This problem creates a vicious spiral, especially for persons who are already vulnerable to mental illness, because a shortage of funds, coupled with anger, discouragement, anxiety, and stress, has a negative impact on their quality of life and the chances of conceiving a child.

In Romania, knowledge about infertility prevention and its causes is basically non-existent. Most couples are unaware that deferring pregnancy has a detrimental effect on future fertility. Lack of knowledge, lack of screening for gynecological problems, high costs of specialist infertility treatments, and associated psychological consequences all contribute to a significant number of infertility cases with restricted access to professional health services. It is advised that public health and medical institutions conduct infertility education services. Additionally, infertility prevention should also be incorporated into other initiatives, such as those targeting the reduction of Sexually Transmitted Infections. Finally, access to genital organ diagnostic imaging such as vaginal ultrasonography should be expanded in order to aid in the early diagnosis of different causes for female infertility [35,36,37].

Besides the limited awareness for infertility treatment and STD prevention in Romania, compared to other European Union countries [38,39,40] due to economic and political factors, the state funding for ART is lower than the average of the EU. However, a further country-wide investigation is required to determine the real numbers of average completed fertility treatments, childlessness, and singleton Romanian families, as the latest UNICEF report warns about Romania [41].

Despite the economic and regulatory obstacles, considerable effort has been made by the Romanian Society for Reproductive Medicine and the Romanian Embryologists Association, as well as by other patient organizations, with the result that the total pregnancy rate obtained is comparable to global statistics. The effectiveness of ART is undeniable, and the number of private clinics in Romania has substantially increased.

Our study had some specific limitations. Every section of the results had a different total number depending on the total number of responses. The number of available correctly collected responses differed from question to question because participating women had different levels of openness, interest, and knowledge when it came to answering each particular question (for example, just 760 women shared their age). Moreover, the amount of time that physicians spent explaining the questions to the patients significantly varied between centers, with different addressability. This may have caused an interviewer bias in some places, which could have affected the answers to the medical questions.

## 5. Conclusions

There are significant disparities between couples that utilize assisted reproductive procedures, particularly in terms of stress levels, family income, and government financial assistance. In Romania, assistance for infertile couples is restricted to a small number of couples that fulfill stringent standards. Therefore, high personal expenses and a generous household income are important in obtaining the desired pregnancy through ART. Additionally, the lack of partner support and advanced female age are also important to consider when counselling couples for ART procedures, since they have a significant impact on the success rate. All of these factors have a detrimental effect on couples’ future chances of becoming pregnant, and couples must work very hard to overcome all of the obstacles.

## Figures and Tables

**Table 1:** Characteristics of women with infertility issues based on the level of stress they faced.

Variables *	Low Stress Exposure	High Stress Exposure	*p*-Value
**Age**			0.017
<35 years (*n* = 437)	169 (38.7%)	268 (61.3%)	
≥35 years (*n* = 290)	138 (47.6%)	152 (52.4%)	
Years until conception, (mean ± SD)	7.87 ± 5.94	8.83 ± 5.94	0.021
**Level of education**			0.011
Primary/Gymnasium/High school (*n* = 177)	61 (34.5%)	116 (65.5%)	
University/Masters/PhD (*n* = 647)	292 (45.1%)	355 (54.9%)	
**Achieved pregnancy after ART**			0.393
No (*n* = 343)	271 (79.1%)	72 (20.9%)	
Yes (*n* = 463)	377 (81.4%)	86 (18.6%)	
**Partner support**			<0.001
No (*n* = 352)	122 (34.7%)	230 (35.3%)	
Yes (*n* = 469)	385 (82.1%)	84 (17.9%)	
**Household income**			<0.001
<1000€ (*n* = 156)	59 (37.8%)	97 (62.2%)	
1000€–1200€ (*n* = 293)	129 (44.3%)	164 (55.7%)	
1200€–1500€ (*n* = 190)	132 (69.5%)	58 (30.5%)	
>1500€ (*n* = 181)	151 (83.4%)	30 (16.6%)	
**Personal expenditures for infertility**			0.010
<4000€ (*n* = 227)	113 (49.8%)	114 (50.2%)	
4000€–12,000€ (*n* = 231)	84 (36.4%)	147 (63.6%)	
12,000€–20,000€ (*n* = 104)	52 (50.0%)	52 (50.0%)	
>20,000€ (*n* = 244)	98 (40.2%)	146 (59.8%)	
**Financial coverage by the government**			<0.001
No coverage (*n* = 667)	204 (30.6%)	463 (69.4%)	
Partial coverage (*n* = 136)	88 (64.7%)	48 (35.3%)	
Full coverage (*n* = 5)	4 (80.0%)	1 (20.0%)	

* Data presented as *n* (frequency), unless specified differently; All frequencies are reported to the total number of respondents on each row.

**Table 2:** Background and expenses faced by infertile couples attempting to achieve pregnancy.

Variables *	Achieved Pregnancy (*n* = 463)	No Pregnancy (*n* = 343)	*p*-Value
**Civil status**			0.131
Married	395 (85.3%)	279 (81.3%)	
Unmarried	68 (14.7%)	64 (18.7%)	
**Intercourse pattern**			0.585
Rarely	63 (13.6%)	52 (15.2%)	
Often	228 (49.2%)	175 (51.0%)	
Frequently	172 (37.2%)	116 (33.8%)	
**Household income**			<0.001
<1000€	50 (10.8%)	106 (30.9%)	
1000€–1200€	153 (33.0%)	140 (40.8%)	
1200€–1500€	129 (27.9%)	61 (17.8%)	
>1500€	131 (28.3%)	36 (10.5%)	
**Cost of ART**			0.022
<1000€	114 (24.6%)	113 (32.9%)	
1000€–2500€	147 (31.8%)	84 (24.6%)	
2500€–4500€	56 (12.1%)	48 (13.9%)	
>4500€	146 (31.5%)	98 (28.6%)	
**Financial coverage by the government**			0.385
No coverage	389 (84.0%)	276 (80.5%)	
Partial coverage	72 (15.6%)	64 (18.7%)	
Full coverage	2 (0.4%)	3 (0.8%)	

* Data presented as *n* (frequency), unless specified differently.

**Table 3:** Multivariate analysis of factors associated with failing to achieve pregnancy through ART.

Factors	OR	95% CI	*p*-Value
**Age**			
<35 ^	1.04	0.63–1.35	0.453
≥35	2.87	1.25–4.01	0.008
**Level of stress**			
Low ^	1.08	0.78–1.21	0.429
High	2.16	1.34–3.07	0.011
**Cost of accessing ART**			
<2500 ^	0.89	0.68–1.07	0.186
≥2500	1.92	1.21–3.05	0.007
**Partner support**			
No ^	1.08	1.01–2.16	0.047
Yes	0.95	0.72–1.23	0.260
**Personal expenditure**			
<12,000	2.03	1.14–3.17	0.024
≥12,000 ^	1.27	1.12–1.58	0.063

OR—Odds ratio; CI—Confidence interval; ^—Reference category.

## Data Availability

The data presented in this study are available on request from the corresponding author.
